# Avaliação da Ablação por Cateter usando Cateter PVAC Gold em Pacientes Idosos com Fibrilação Atrial

**DOI:** 10.36660/abc.20240306

**Published:** 2024-07-11

**Authors:** José Carlos Pachon-M, Tomas G. Santillana-P, Enrique I. Pachon-M

**Affiliations:** 1 Faculdade de Medicina Universidade de São Paulo São Paulo SP Brasil Faculdade de Medicina da Universidade de São Paulo, São Paulo, SP – Brasil; 2 Hospital do Coração São Paulo SP Brasil Hospital do Coração, São Paulo, SP – Brasil

**Keywords:** Cateteres, Ablação por Cateter, Fibrilação Atrial

A fibrilação atrial (FA) representa um dos desafios mais significativos na prática cardiológica contemporânea, especialmente em pacientes idosos, onde a complexidade do tratamento é amplificada pelas comorbidades prevalentes e pela fragilidade desta população. Nesse contexto, é notável o estudo “Estudo Randomizado Comparando a Ablação por Cateter com o PVAC Gold vs. Tratamento com Fármacos Antiarrítmicos em Pacientes Idosos com Fibrilação Atrial Sintomática”, recentemente publicado no ABC Cardiol por Martins et al.^1^ Ele oferece *insights* cruciais sobre opções terapêuticas, riscos e benefícios para esses pacientes. Além disso, este artigo é liderado por uma equipe com notável experiência internacional em ablação de FA, enfrentando os desafios com julgamento clínico astuto e sendo pioneira em diversos procedimentos.

Este grupo de pacientes frequentemente polimedicados inspira constantemente a busca por terapias que reduzam a dependência medicamentosa e melhorem a qualidade de vida. A perspectiva de eliminar a FA através da ablação e reduzir múltiplos medicamentos é altamente atraente.^2,3^

O estudo compara a eficácia da ablação por cateter utilizando o cateter Medtronic PVAC Gold, que não é irrigado, com o tratamento convencional com medicamentos antiarrítmicos. Os resultados clínicos de ambas as estratégias mostraram melhorias na qualidade de vida dos pacientes, embora sem diferenças estatisticamente significativas nos resultados clínicos primários ou secundários após um ano de acompanhamento.

O cateter PVAC Gold é uma obra-prima da engenharia com design inovador e alto perfil de segurança e praticidade. Contudo, a sua principal limitação técnica é a falta de irrigação. A este respeito, o artigo fornece informações notáveis através da realização de ressonância magnética e endoscopia digestiva pós-ablação, destacando complicações frequentemente negligenciadas em muitos ensaios: tromboembolismo e lesões esofágicas subclínicas.

Um dos achados mais preocupantes foi a taxa de 25% de embolização cerebral em pacientes tratados com cateter PVAC Gold. Essa elevada taxa tromboembólica pode ser atribuída à falta de irrigação do cateter, permitindo maior geração de calor e, consequentemente, maior risco de formação de coágulos. Além disso, vale ressaltar que os autores encontraram lesões esofágicas apesar do uso de termômetro esofágico.^4^ Esses achados geralmente não são supervisionados em muitos estudos.

Além disso, a reintervenção em 30% do grupo de ablação também suscita preocupações. Isto indica uma limitação na eficácia do procedimento inicial, potencialmente relacionada às limitações do cateter. No entanto, todas as técnicas de ablação de FA apresentam taxas de reintervenção variáveis, ocasionalmente excedendo 30%. Apesar dos avanços significativos, um dos fatores críticos que limitam o sucesso da ablação é a natureza complexa e multifacetada da fisiopatologia da FA.^5-8^ Esta complexidade requer uma abordagem abrangente e multidisciplinar para compreender completamente e abordar eficazmente os mecanismos subjacentes da FA.

No geral, o estudo demonstra que, embora a ablação com PVAC Gold não tenha reduzido significativamente a taxa de recorrência de FA em comparação com o tratamento medicamentoso, foi associada a uma menor progressão para formas persistentes da arritmia e a uma redução no uso de amiodarona, um resultado notável, dado o perfil de efeitos colaterais da amiodarona e os desafios no manejo desses efeitos em pacientes idosos.^1^

A análise dos desfechos secundários, como qualidade de vida relacionada à saúde, não mostrou diferenças significativas entre os grupos, levantando questões importantes sobre a percepção do paciente quanto à melhora dos sintomas. Melhorar a qualidade de vida é fundamental na avaliação de qualquer intervenção terapêutica. A ausência de diferença neste parâmetro sugere que a decisão entre ablação e tratamento farmacológico deve ser altamente individualizada, considerando não apenas a eficácia clínica, mas também as preferências e expectativas do paciente.

Considerando essas descobertas, várias preocupações são dignas de nota:

Reavaliação da Tecnologia: A ablação com cateteres irrigados, que apresentou menores taxas de complicações nas metanálises, deve ser considerada uma alternativa mais segura, pois auxilia na prevenção do tromboembolismo.

Termômetros esofágicos devem sempre ser considerados.

As complicações subclínicas, como embolias cerebrais, sugerem a necessidade de protocolos de monitoramento pós-procedimento mais rigorosos, incluindo ressonância magnética e endoscopia digestiva em casos suspeitos.

O estudo ressalta a importância da pesquisa clínica contínua focada na segurança e eficácia da ablação em pacientes idosos. A ablação da FA está evoluindo rapidamente e novos conhecimentos sobre a sua fisiopatologia revelam tendências emergentes. Foi demonstrado que a ablação da interface neuromiocárdica pode reduzir drasticamente a recorrência após o procedimento, beneficiando pacientes de todas as idades.^6,9^

Os pacientes devem ser totalmente informados sobre os riscos e benefícios das diferentes opções de tratamento, com uma discussão transparente sobre possíveis complicações e expectativas de sucesso do tratamento.

Assim, este notável estudo^1^ fornece informações valiosas para o manejo da FA em pacientes idosos, uma população que desafia as práticas convencionais devido à sua complexidade e fragilidade.

## References

[1] Martins LCB, Pisani CF, Dorfman FK, Darrieux FCC, Wu TC, Ferraz AP, Hachul DT (2024). Estudo Randomizado Comparando a Ablação por Cateter com o PVAC Gold vs. Tratamento com Fármacos Antiarrítmicos em Pacientes Idosos com Fibrilação Atrial Sintomática. Arq Bras Cardiol.

[2] Li W, Wang YC, Tiwari N, Di Biase L (2022). Amiodarone is Associated with Increased Short-term Mortality in Elderly Atrial Fibrillation Patients with Preserved Ejection Fraction. J Interv Card Electrophysiol.

[3] França MRQ, Morillo CA, Carmo AAL, Mayrink M, Miranda RC, Naback ADN (2024). Efficacy and Safety of Catheter Ablation for Atrial Fibrillation in Elderly Patients: A Systematic Review and Meta-analysis. J Interv Card Electrophysiol.

[4] Scanavacca M (2016). Current Atrial Fibrillation Ablation: An Alert for the Prevention and Treatment of Esophageal Lesions. Arq Bras Cardiol.

[5] Nattel S, Harada M (2014). Atrial Remodeling and Atrial Fibrillation: Recent Advances and Translational Perspectives. J Am Coll Cardiol.

[6] Pachon M JC, Pachon M EI, Pachon M JC, Lobo TJ, Pachon MZ, Vargas RN (2004). A New Treatment for Atrial Fibrillation Based on Spectral Analysis to Guide the Catheter RF-ablation. Europace.

[7] Scanavacca M, Sosa E (2009). Catheter Ablation Techniques for Selective Cardiac Autonomic Denervation to Treat Patients with Paroxysmal Atrial Fibrillation. Heart Rhythm.

[8] Rivarola EW, Scanavacca M, Ushizima M, Cestari I, Hardy C, Lara S (2011). Spectral Characteristics of Atrial Electrograms in Sinus Rhythm Correlates with Sites of Ganglionated Plexuses in Patients with Paroxysmal Atrial Fibrillation. Europace.

[9] Pachon-M EI, Clark J, Lobo TJ, Pachon CT, Higuti C, Pachon MZ (2023). Impact of Cardioneuroablation with Vagal Denervation Confirmed by Vagus Nerve Stimulation on Pulmonary Vein Isolation for Atrial Fibrillation Catheter Ablation. J Atr Fibrillation Electrophysiology.

