# Supplementary material for: Avaliação da Ablação por Cateter usando Cateter PVAC Gold em Pacientes Idosos com Fibrilação Atrial
Source: Arq Bras Cardiol. 2024 Jul 11;121(6):e20240306. [Article in Portuguese] doi: 10.36660/abc.20240306 (PMC12080615; doi:10.36660/abc.20240306)
Supplement: Translation

# Translation: Evaluation of Catheter Ablation Using the PVAC Gold Catheter in Elderly Patients with Atrial Fibrillation

**DOI:** 10.36660/abc.20240306i

**Authors:** José Carlos Pachon-M, Tomas G. Santillana-P, Enrique I. Pachon-M

**Affiliations:** 1 Brazil Faculdade de Medicina da Universidade de São Paulo, São Paulo, SP – Brazil; 2 Brazil Hospital do Coração, São Paulo, SP – Brazil

**Keywords:** Catheters, Catheter Ablation, Atrial Fibrillation

Atrial fibrillation (AF) represents one of the most significant challenges in contemporary cardiological practice, especially in elderly patients, where the complexity of treatment is amplified by prevalent comorbidities and the fragility of this population. In this context, the study “Randomized Study Comparing Radiofrequency Ablation with the PVAC Gold System vs. Antiarrhythmic Drugs in Elderly Patients with Symptomatic Atrial Fibrillation” recently published in the ABC Cardiol by Martins et al.,^1^ is remarkable. It offers crucial insights into therapeutic options, risks, and benefits for these patients. Additionally, this article is led by a team with remarkable international experience in AF ablation, navigating the challenges with astute clinical judgment having been a pioneer in several procedures.

This group of frequently polymedicated patients constantly inspires the search for therapies that reduce medication dependence and enhance quality of life. The prospect of eliminating AF through ablation and reducing multiple medications is highly appealing.^2,3^

The study compares the efficacy of catheter ablation using the Medtronic PVAC Gold catheter, which is non-irrigated, with conventional treatment using antiarrhythmic drugs. The clinical outcomes of both strategies showed improvements in patients’ quality of life, though without statistically significant differences in primary or secondary clinical outcomes after one year of follow-up.

The PVAC Gold catheter is a masterpiece of engineering with an innovative design and high safety and practicality profile. However, its main technical limitation is the lack of irrigation. In this regard, the article provides notable information by performing MRI and digestive endoscopy post-ablation, highlighting often overlooked complications in many trials: thromboembolism and sub-clinical esophageal lesions.

One of the most concerning findings was the 25% rate of cerebral embolization in patients treated with the PVAC Gold catheter. This high thromboembolic rate can be attributed to the lack of catheter irrigation, allowing higher heat generation and, consequently, an increased risk of clot formation. Additionally, it is noteworthy that the authors found esophageal lesions despite using an esophageal thermometer.^4^ These findings are commonly unsupervised in many trials.

Furthermore, the reintervention in 30% of the ablation group also raises concerns. This indicates a limitation in the initial procedure’s efficacy, potentially related to the catheter’s limitations. However, all AF ablation techniques have variable reintervention rates, occasionally exceeding 30%. Despite significant advancements, one of the critical factors limiting ablation success is the complex and multifaceted nature of AF pathophysiology.^5-8^ This complexity requires a comprehensive and multidisciplinary approach to fully understand and effectively address the underlying mechanisms of AF.

Overall, the study demonstrates that although ablation with PVAC Gold did not significantly reduce the recurrence rate of AF compared to drug treatment, it was associated with less progression to persistent forms of the arrhythmia and a reduction in the use of amiodaron, a notable result, given amiodarone’s side effect profile and the challenges in managing these effects in elderly patients.^1^

The analysis of secondary outcomes, such as health-related quality of life, showed no significant differences between the groups, raising important questions about the patient’s perception of symptom improvement. Improving quality of life is critical in evaluating any therapeutic intervention. The absence of difference in this parameter suggests that the decision between ablation and pharmacological treatment should be highly individualized, considering not only clinical efficacy but also patient preferences and expectations.

Considering these findings, several concerns are worth noting:

Reevaluation of Technology: Ablation with irrigated catheters, which have shown lower complication rates in meta-analyses, should be considered a safer alternative as it helps thromboembolism prevention.

Esophageal Thermometers should always be considered.

The subclinical complications, such as cerebral embolisms, suggest the need for stricter post-procedure monitoring protocols, including MRI and digestive endoscopy in suspected cases.

The study underscores the importance of ongoing clinical research focused on the safety and efficacy of ablation in elderly patients. AF ablation is rapidly evolving, and new insights into its pathophysiology are revealing emerging trends. It has been demonstrated that ablation of the neuro-myocardial interface can drastically reduce recurrence following the procedure, benefiting patients of all ages.^6,9^

Patients must be fully informed about the risks and benefits of different treatment options with a transparent discussion about potential complications and treatment success expectations.

Thus, this notable study^1^ provides valuable insights for managing AF in elderly patients, a population that challenges conventional practices due to its complexity and fragility.
